# Two-Level Optical Coherence Tomography Scheme for Suppressing Spectral Saturation Artifacts

**DOI:** 10.3390/s140813548

**Published:** 2014-07-25

**Authors:** Chiung-Ting Wu, Meng-Tsan Tsai, Cheng-Kuang Lee

**Affiliations:** 1 Graduate Institute of Medical Mechatronics, Chang Gung University, 259, Wen-Hwa 1st Road, Kwei-Shan, Tao-Yuan 33302, Taiwan; E-Mail: CTWu@mail.cgu.edu.tw; 2 Department of Electrical Engineering, Chang Gung University, 259, Wen-Hwa 1st Road, Kwei-Shan, Tao-Yuan 33302, Taiwan; E-Mail: mttsai@mail.cgu.edu.tw

**Keywords:** OCT, noise in imaging system, real-time

## Abstract

We demonstrate a novel method for reducing saturation artifacts in spectral-domain optical coherence tomography (SD-OCT) systems. This method is based on a two-level SD-OCT system with a dual-line charge-coupled device (CCD) camera. We compensate the saturated signal detected by the first line using the unsaturated signal detected by the second line. The Fourier transform of the compensated spectrum shows effective suppression of saturation artifacts. This method was also successfully performed on phantom material and skin on a human finger. Our method causes neither back-scattering power loss nor signal-to-noise ratio (SNR) degradation. The only difference between the traditional system and our two-level system is our utilization of the dual-line CCD camera; no additional devices or complex designs are needed.

## 1. Introduction

Optical coherence tomography (OCT) has been demonstrated in various biomedical applications. In recent years, the successful development of Fourier-domain OCT (FD-OCT) was very beneficial for certain applications, thus reducing the number of traditional time-domain OCT (TD-OCT) users. Though FD-OCT has many advantages over TD-OCT, FD-OCT is still prone to several kinds of image artifacts. The kind of artifacts will be initially noticed is the mirror image, which is due to the lacking imaginary part of the OCT signal. Several methods have been reported for reducing these artifacts, and most use a technique that adds an additional phase shift to the OCT signal. By detecting the phase shift of the real image and mirror image between A-mode scans, we can suppress the mirror image from the real image [1–4].

When introducing the OCT technique in biomedical applications, motion artifacts became problematic. As we scan a living creature, any uncontrollable trembling will blur the signal. If you take a photo of a flying ball, the blur on the picture is a demonstration of this type of motion artifacts. Since this kind of artifacts is prevailing in biomedical applications, several approaches to solving the motion artifacts have already been published [5–7].

In addition to motion artifacts, autocorrelation introduces another kind of typical artifacts in biomedical applications. If the sample has a strong scattering layer, it may cause image artifacts due to strong autocorrelation; in biomedical applications, the cover glass on slides may cause scattering. The basic principles of OCT are to analyze strong fluctuation signals produced by the interference between the light received at the sample arm and reference arm. However, if the light backscattering from the sample also produces strong interference, this unwanted signal will become artifacts on the OCT image. To reduce the autocorrelation artifacts, several methods have been proposed [8,9].

The FD-OCT can be classified into either swept-source OCT (SS-OCT) or spectral-domain OCT (SD-OCT) based on the detection scheme. The SS-OCT detector is typically a balanced detector, while the SD-OCT detector is a line-scan camera. However, if the signal fluctuation exceeds the dynamic range of the detector, it will lead to saturation artifacts. The real shape of the signal may be deformed by error detection, which “chops off the head” of the real signal, leading to artifacts on the OCT images. Furthermore, due to the line-scan camera detector scheme, saturation artifacts are more significant in SD-OCT. The dynamic range limitation on data acquisition with the line-scan camera is usually 8–12 bits, which is the primary reason for the saturation artifacts.

Unlike other artifacts, there are no developed methods for solving saturation artifacts. Recently, the reduction of saturation artifacts has gained more interest. Huang *et al.* [10] attempted to fix saturation artifacts by interpolation. First, they identified A-mode scan lines with saturation points. Then, they replaced these lines by interpolating the adjacent normal A-mode scan lines on the OCT image. This method could be effective if saturation scan lines always appear separately. However, if there are too many continuous saturation A-mode scans, this method is very imprecise and may cause additional errors during OCT image analysis.

## 2. Experimental Section

**Figure 1.**
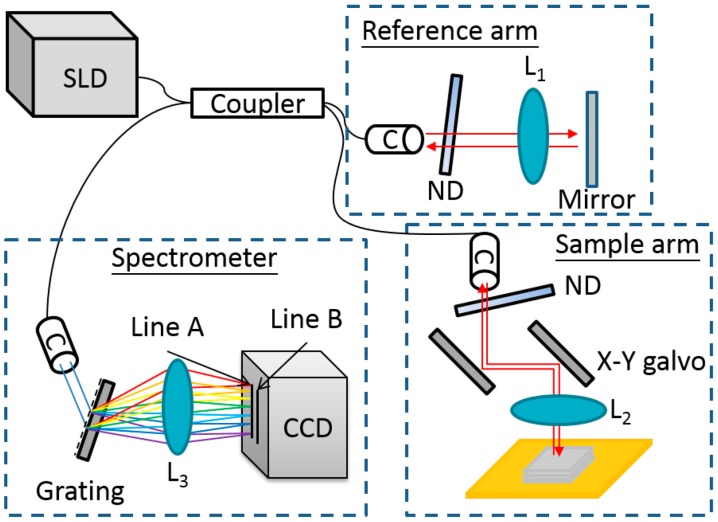
Scheme for the two-level spectral-domain optical coherence tomography (SD-OCT) system with a fiber coupler (C), dual-line scan camera (CCD), lens (L), neutral density filter (ND), and superluminescent diode (SLD). The focus line of lens 3 (L3) is at Line A, and the line slightly shifted from the focus of lens 3 is Line B.

We demonstrate the first two-level SD-OCT system, shown in Figure 1, which can reduce the saturation artifacts. In our system, the center wavelength of the superluminescent diode (D890-HP, Superlum) is 890 nm. The spectrometer is constructed with a grating (HD 1800 1/mm, Wasatch Photonics) and a dual-line CCD camera (Spl4096-140km, Basler). The image acquisition device (PCIe-1433, National Instruments) is set at its maximum of 12-bits. When saturation occurs, the signal intensity reaches a value of 4095 (arbitrary units). The key point of the two-level SD-OCT system is the CCD camera, which has “dual line” acquisition mode. The camera can simultaneously acquire signals from line A and line B. We adjust line A to match the focus of the lens before the CCD camera. Thus, line A is the main detector in the system. Though line B is not in focus, it can also acquire a weak signal.

**Figure 2.**
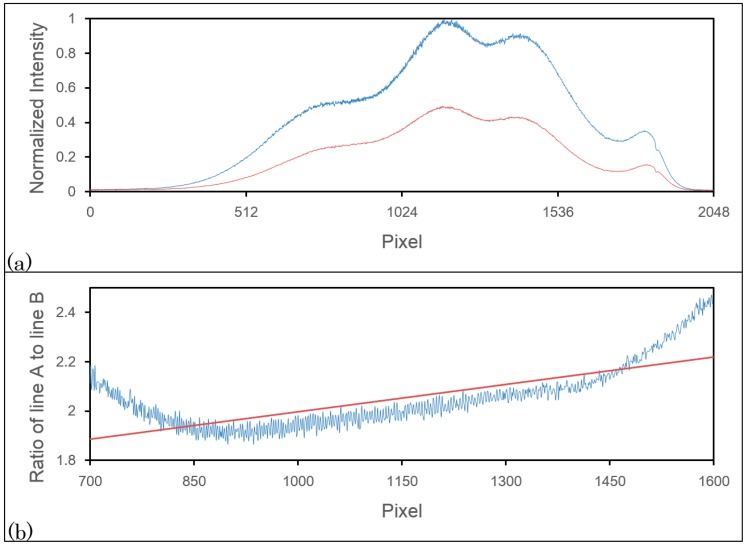
(**a**) The light source spectra received by line A (blue) and line B (red); the red line intensity is about half that of the blue line, and the shapes of the two lines are very similar; (**b**) The ratio of spectra received by line A to that by line B from the 700th pixel to 1600th pixel (blue), and its linear fitting line (red).

Figure 2a shows the light source spectra received by line A and line B. The power detected by line B is about half that of line A, and the shapes of the detected signals are very similar. Thus, we can use the signal detected by line B to compensate for the saturation point on line A. In normal situations, because the power projected on line A will not significantly exceed the saturation limit of line A, saturation should not occur on line B.

Suppose real numbers A_n_ and B_n_ represent the detected signals of the nth pixel on line A and line B, respectively, and r_n_ is defined as the ratio of A_n_ to B_n_. We assume that the saturation problem occurs on line A between pixels (*k* + 1) and (*k* + *m*), such that the values of A_(k+1)_ to A_(k+m)_ are 4095. To estimate the real value of A_n_ (the real signal without saturation), we assume there is a nearly linear relationship between *n* and r_n_ for *n* between *k* to *k* + *m* + 1. Thus, from the linear interpolation formula for *l* from 1 to *m*, we can write

(1) $$r_{k+l}=\frac{l}{m+1}(r_{k+m+1}-r_{k})+r_{k}$$

where *k* represents the number of normally detected pixels before the saturated pixels, and *m* is the number of saturated pixels. Because r_n_ is the ratio of A_n_ to B_n_, we can rewrite Equation (1) as

(2) $$A_{k+l}^{'}=B_{k+l}\frac{l}{m+1}(\frac{A_{k+m+1}}{B_{k+m+1}}-\frac{A_{k}}{B_{k}})+\frac{A_{k}}{B_{k}}$$

where A'_k+l_ is the estimated real signal that could be detected by line A if there were no saturation problems. To verify the linear relationship, we calculated the ratio of spectra in Figure 2a received by line A to that by line B and showed in Figure 2b. One may notice that the pixel range shown in Figure 2b is only from 700 to 1600. As we know, that only when the intensity is strong enough would cause saturated, so here we just verified the pixels whose normalized intensity are greater than 0.5. The residue of linear fitting in Figure 2b with least square method is 0.007391. However, based on our experimental observations, the number of sequential saturated pixel is usually less than 10; hence, we performed the least square method every 10 pixels. The average of residue within 10 pixels is 0.0004159, which is much smaller than that under the case of pixels within 700 to 1600. Thus, we can see that the assumption of linearity is reliable, but not perfect, so we can predict that there may still be some artifacts after our compensation method.

**Figure 3.**
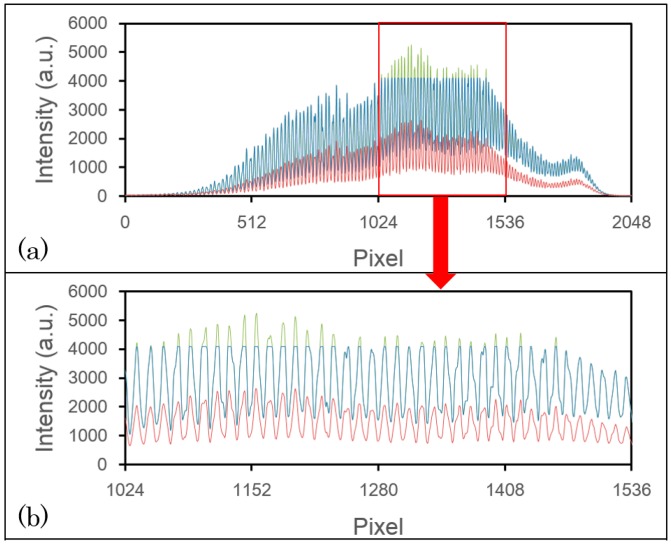
The A-mode scan spectra of a sample consisting of 3 M Post-it Note paper with five layers of semi-opaque tape. (**a**) The saturated spectrum detected by line A (blue), the unsaturated spectrum detected by line B (red), and the compensated spectrum (green); (**b**) The enlarged spectrum indicated by the red square in (a).

To demonstrate this compensation method, we used 3 M Post-it Note paper with five layers of semi-opaque tape as a phantom material. Figure 3 shows one of the saturated spectra. In Figure 3a, the blue line represents the signal detected by line A, and the red line represents the line B signal. As we can see, there is a cut off at 4095 in the blue line, which occurs between the pixels 1024 and 1536. No saturation problem occurs in the red line, so we can use the red line to compensate for the blue line. The green line is the outcome of the compensation, and Figure 3b shows the enlarged spectra of the saturated area. From the peaks without saturation in the blue line, we can see that the peak shapes are very similar to the corresponding peaks in the red line. After applying our compensation method, the peak shapes of the green line are also very similar to the corresponding ones on the red line. The maximum of the green line is approximately 5200, which exceeds the maximum of the blue line by about 1100. Thus, Figure 3 demonstrates that even though the power on line A is about five-fourths of the CCD maximum, there are no saturation effects on line B; therefore, our method can compensate for the signal loss on line A.

## 3. Results and Discussion

**Figure 4.**
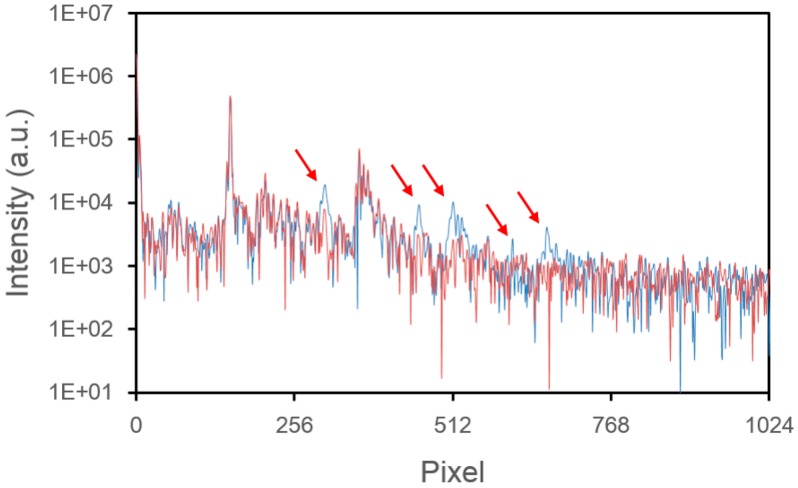
Fourier transforms of the saturated (blue) and compensated (red) spectra from Figure 3.

To demonstrate the effect of our compensation method, we compare the Fourier transform of the signals shown in Figure 3 before and after compensation (Figure 4). The red arrows indicate locations where saturation artifacts occur (blue line) and the result of compensation (red line). We see the saturation artifacts are obviously suppressed.

**Figure 5.**
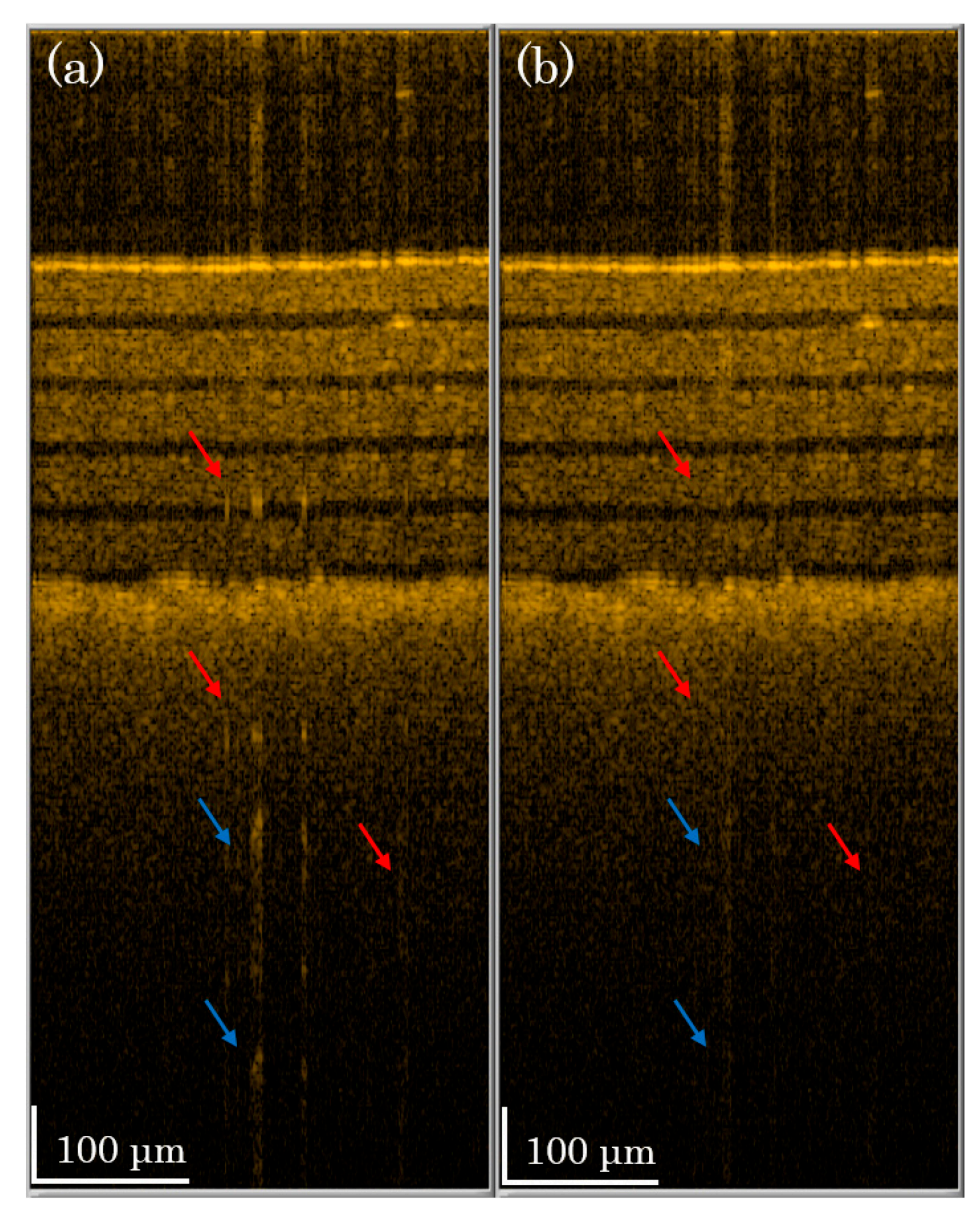
The B-mode scan image of 3 M Post-it Note paper with five layers of tape (**a**) without saturation artifact correction and (**b**) with the two-level compensation method.

In Figure 5, we demonstrate the OCT image of the phantom sample of Figure 4. One B-mode scan image consists of 500 A-mode scans, and the A-mode scan shown in Figure 4 is the 250th line in Figure 5. In Figure 5a, we see many artifacts (indicated by arrows) in the image due to saturation problems. After performing our compensation method, shown in Figure 5b, most of the artifacts are suppressed (red arrows). One can see that some artifacts are not completely suppressed (blue arrows), because the assumption of linearity is not perfect. However, our method can significantly reduce the saturation artifacts.

**Figure 6.**
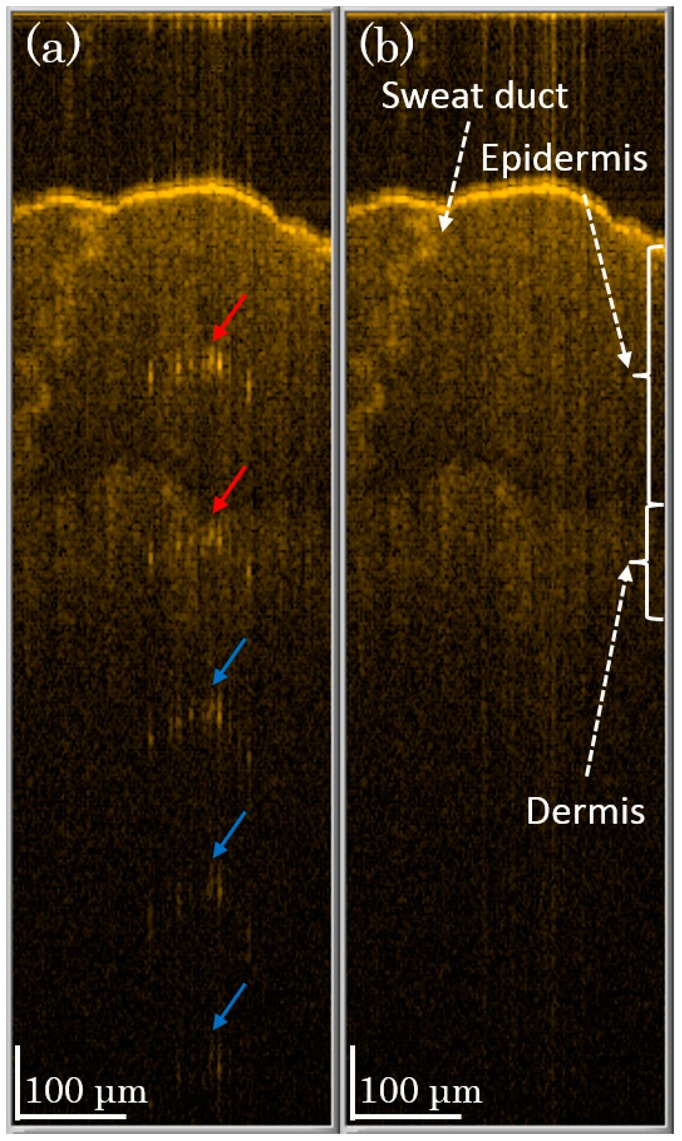
B-mode scan images of the skin of a human finger (**a**) without saturation artifact correction and (**b**) with the two-level compensation method.

To demonstrate the performance of our method on living tissue, we scan *in vivo* skin on a human finger. In Figure 6a, the saturation artifacts (indicated by arrows) are caused by strong scattering at the skin surface. The first two artifacts (red arrows) may disturb the image analysis. However, in Figure 6a, we see that the first two artifacts are suppressed with our method, and we see a clear tissue image. Though the last three artifacts (blue arrows) do not affect the tissue image, these artifacts are still well suppressed after our method is applied. Therefore, in Figure 5 and Figure 6, we demonstrate that our method can reduce saturation problems on not only phantom materials but also tissue *in situ*.

## 4. Conclusions

In summary, we have demonstrated a two-level SD-OCT system with a method to compensate for saturated spectra. This method can suppress saturation artifacts without any back-scattering power loss or SNR degradation. Further, our method can be executed with only one CCD camera; no additional devices are needed.
